# 669. The clinical effectiveness of Fidaxomicin compared to Vancomycin in the treatment of *Clostridioides difficile* infection, a single center real-world experience

**DOI:** 10.1093/ofid/ofad500.731

**Published:** 2023-11-27

**Authors:** Majd Alsoubani, Jennifer K Chow, Angie Mae Rodday, David Kent, David R Snydman

**Affiliations:** Tufts Medical Center, Boston, Massachusetts; Division of Geographic Medicine and Infectious Diseases, Tufts Medical Center, Boston, MA; Tufts Medical Center, Boston, Massachusetts; Predictive Analytics and Comparative Effectiveness (PACE) Center, Institute for Clinical Research and Health Policy Studies, Tufts Medical Center, Boston, Massachusetts; Tufts Medical Center, Boston, Massachusetts

## Abstract

**Background:**

*Clostridioides difficile* infection (CDI) recurs in up to 25% of patients who achieve initial response to treatment. Therefore, the goal of treatment is to establish initial cure and prevent relapses. In this study, we examine the clinical effectiveness of fidaxomicin compared to vancomycin in a real-world setting.

**Methods:**

This was a retrospective study conducted between 2011 and 2021 among patients who were treated with vancomycin or fidaxomicin for CDI at Tufts Medical Center. Patients were allocated to each treatment group if they received 72 hours of the agent. Patients less than 18 years, those who did not receive any treatment for CDI those treated with metronidazole only or fecal microbiota transplant were excluded. The primary study outcome was a composite of failure to achieve clinical cure within 72 hours of treatment initiation, relapse within 30 days following completion of initial treatment or death related to CDI. A secondary outcome of combined 30 and 90 day relapse was evaluated. A time to event analysis using a cause specific Cox proportional hazards model comparing the rate of the composite outcome in the two groups was conducted, accounting for the competing risk of death from other causes. Multiple imputation was used for missing variables but not the outcome.

**Results:**

A total of 637 patients were diagnosed with CDI and able to be analyzed. There were 550 patients who received vancomycin compared to 87 patients who received fidaxomicin. Patients who received fidaxomicin were more likely to be female, have history of prior CDI and were on dialysis compared to those who received vancomycin (Table 1). The composite outcome occurred in 13 (14.9%) patients in the fidaxomicin group compared to 139 (25.3%) in the vancomycin group (Table 2). The adjusted hazard ratio for the composite outcome was significantly lower in the fidaxomicin group compared to vancomycin (HR 0.5, 95% CI 0.3-0.9) (Table 3). The adjusted risk of the secondary outcome, 30 and 90 day relapse, was reduced by 34% in the fidaxomicin group compared to vancomycin (HR 0.34, 95% CI 0.15-0.77).

**Figure:**
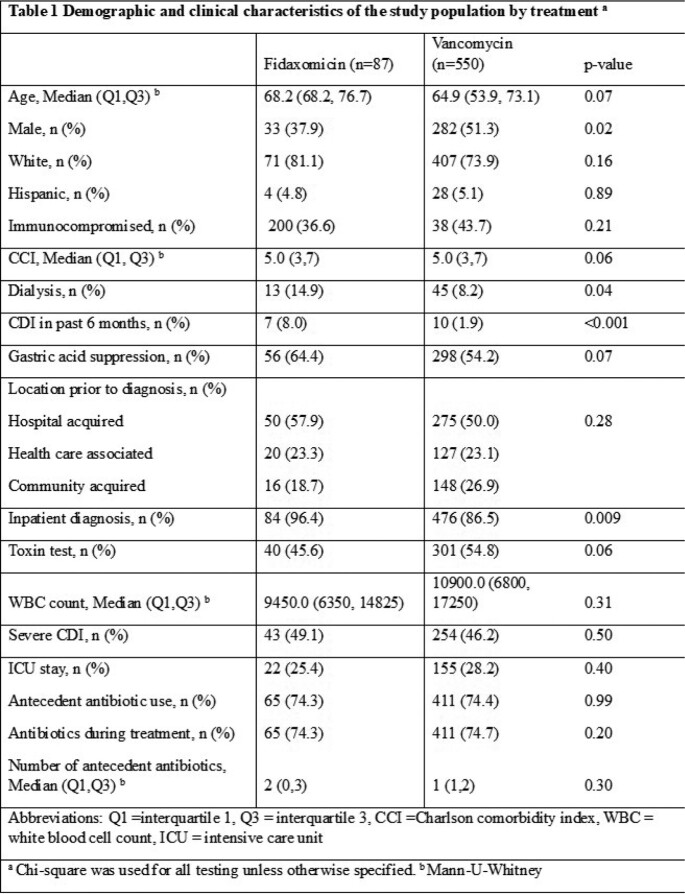

**Figure:**
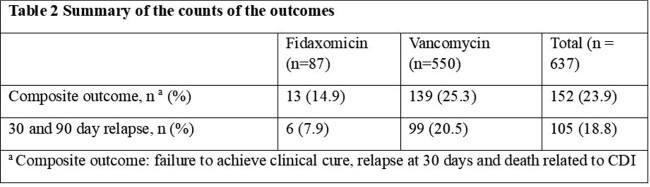

**Figure:**
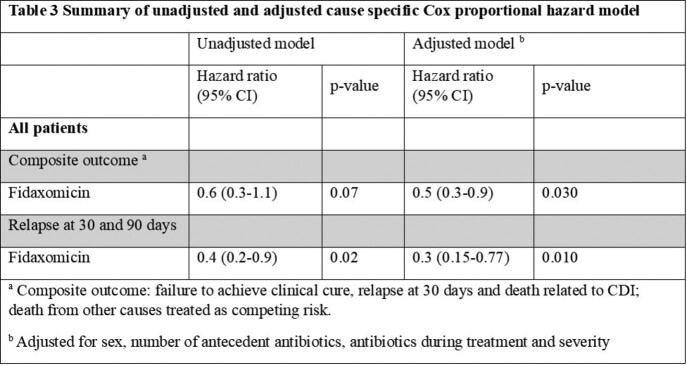

**Conclusion:**

Our analysis demonstrates that the use of fidaxomicin was associated with reduced hazard of CDI poor treatment outcomes. This study describes real world experience using fidaxomicin in patients with CDI.

**Disclosures:**

**Jennifer K. Chow, MD, MS**, Kamada: Grant/Research Support|Merck: Grant/Research Support|Moderna: DSMB **David Kent, M.D., M.S.**, W.L. Gore: Grant/Research Support **David R. Snydman, MD**, Merck: Advisor/Consultant|Merck: Grant/Research Support|Prolacta: Advisor/Consultant|Prolacta: Grant/Research Support|Seres therapeutics: Advisor/Consultant|Seres Therapeutics: Grant/Research Support|Summit Therapeutics: Grant/Research Support

